# VMDU-net: a dual encoder multi-scale fusion network for polyp segmentation with Vision Mamba and Cross-Shape Transformer integration

**DOI:** 10.3389/frai.2025.1557508

**Published:** 2025-06-18

**Authors:** Peng Li, Jianhua Ding, Chia S. Lim

**Affiliations:** ^1^School of Computing & Technology, Asia Pacific University of Technology & Innovation, Lebuhraya Bukit Jalil, Taman Teknologi Malaysia, Bukit Jalil, Kuala Lumpur, Malaysia; ^2^Gansu Provincial Tumor Hospital, Lanzhou, China

**Keywords:** polyp segmentation, Mamba, Transformer, feature fusion, medical image segmentation

## Abstract

**Introduction:**

Rectal cancer often originates from polyps. Early detection and timely removal of polyps are crucial for preventing colorectal cancer and inhibiting its progression to malignancy. While polyp segmentation algorithms are essential for aiding polyp removal, they face significant challenges due to the diverse shapes, unclear boundaries, and varying sizes of polyps. Additionally, capturing long-range dependencies remains difficult, with many existing algorithms struggling to converge effectively, limiting their practical application.

**Methods:**

To address these challenges, we propose a novel Dual Encoder Multi-Scale Feature Fusion Network, termed VMDU-Net. This architecture employs two parallel encoders: one incorporates Vision Mamba modules, and the other integrates a custom-designed Cross-Shape Transformer. To enhance semantic understanding of polyp morphology and boundaries, we design a Mamba-Transformer-Merge (MTM) module that performs attention-weighted fusion across spatial and channel dimensions. Furthermore, Depthwise Separable Convolutions are introduced to facilitate multi-scale feature extraction and improve convergence efficiency by leveraging the inductive bias of convolution.

**Results:**

Extensive experiments were conducted on five widely-used polyp segmentation datasets. The results show that VMDU-Net significantly outperforms existing state-of-the-art methods, especially in terms of segmentation accuracy and boundary detail preservation. Notably, the model achieved a Dice score of 0.934 on the Kvasir-SEG dataset and 0.951 on the CVC-ClinicDB dataset.

**Discussion:**

The proposed VMDU-Net effectively addresses key challenges in polyp segmentation by leveraging complementary strengths of Transformer-based and Mamba-based modules. Its strong performance across multiple datasets highlights its potential for practical clinical application in early colorectal cancer prevention.

**Code availability:**

The source code is publicly available at: https://github.com/sulayman-lee0212/VMDUNet/tree/4a8b95804178511fa5798af4a7d98fd6e6b1ebf7.

## 1 Introduction

**Figure 1:**
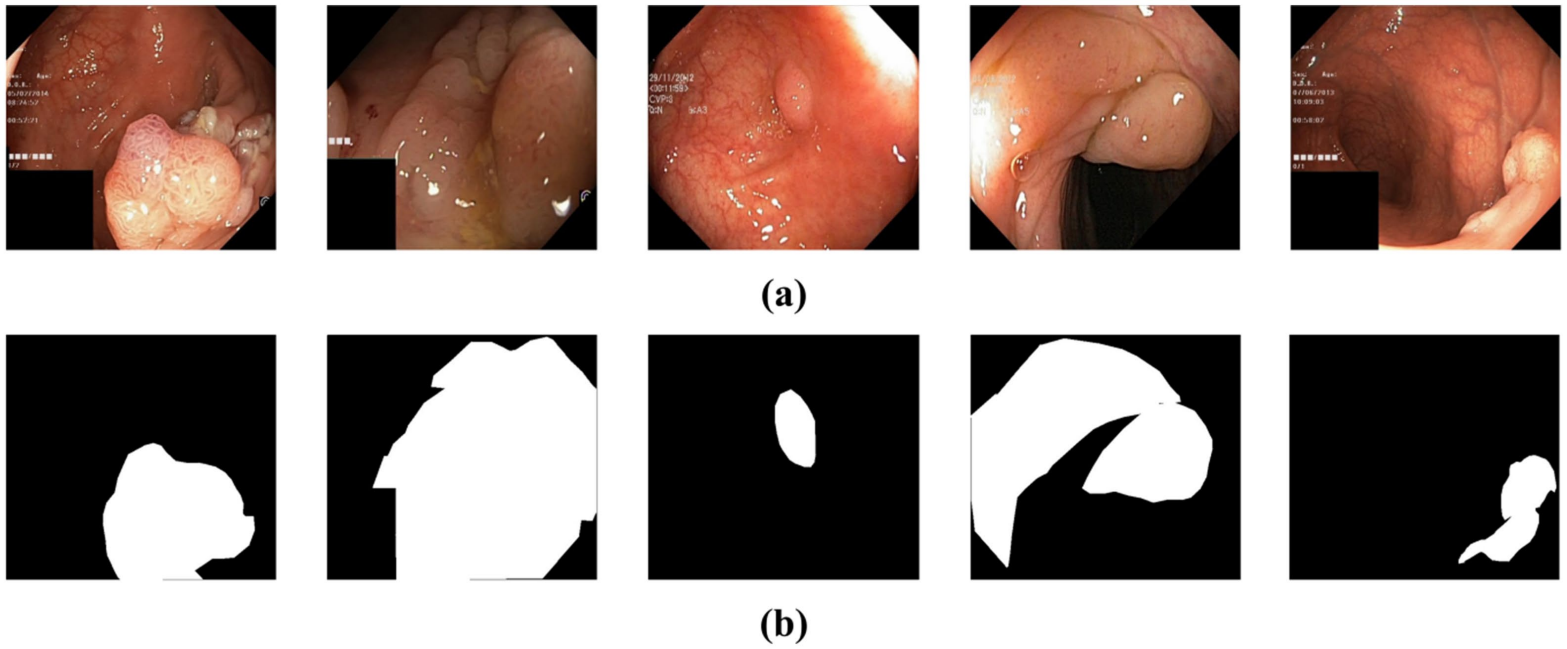
**(a)** The original polyp images exhibit varying sizes, blurred boundaries, and weak contrast between the lesion and the background. **(b)** Ground truth map for polyp segmentation.

In terms of incidence rates, colorectal cancer (CRC) is the third most common malignant tumor in the world (Gupta and Mishra, 2024). Therefore, preventing CRC through regular screening and the removal of precancerous lesions, such as colorectal adenomas, has become a crucial focus for public health systems worldwide. Colonoscopy, as a widely-used screening method for CRC, allows for the identification of the location and surface characteristics of colorectal polyps, enables physicians to remove polyps before their development into cancer, and thus achieves a preventive effect. As shown by studies in Haggar and Boushey (2009), early screening can reduce CRC incidence by up to 30%. Therefore, accurate polyp segmentation is essential for improving screening efficiency and reducing missed diagnoses. However, the task brings multiple challenges. First, polyps exhibit significant morphological diversity and vary in size, color, and texture. Second, in colonoscopy images, the boundaries between polyps and surrounding tissues are often blurred and lack distinct contrast, increasing the segmentation difficulty. These factors make automated segmentation prone to errors or omissions, limiting the effectiveness of current polyp segmentation algorithms. To realize the early detection and prevention of CRC, it is of critical clinical importance to develop an automated segmentation method capable of accurately and efficiently detecting polyps (Jia et al., 2019) (see Figure 1).

Typically, traditional polyp segmentation techniques rely on surface features such as shape, texture, and simple clustering for initial image segmentation (Sasmal et al., 2022). However, compared to deep learning algorithms, these methods often struggle to achieve high segmentation accuracy. With the rapid development of deep learning, convolutional neural networks (CNNs) have become powerful tools that can represent more complex features and significantly improve the performance of colorectal polyp segmentation. Ronneberger et al. (2015) proposed U-Net, which consists of a downsampling (encoder) part and an upsampling (decoder) part, and forms a “U”-shaped structure. The encoder is in charge of feature extraction, while the decoder generates high-resolution segmentation maps. Zhou et al. (2018) introduced U-Net++, an improved version of U-Net for enhancing the accuracy of medical image segmentation. U-Net++ incorporates nested skip connections and deep supervision mechanisms, which improve information flow between different feature levels and optimize the fusion of multi-resolution features. Given that U-Net++ architecture excels in handling fine details and blurred boundaries, it is widely applicable to complex image segmentation tasks. Diakogiannis et al. (2020) proposed ResUNet, which combines residual connections with multi-scale feature extraction to mitigate the vanishing gradient problem and enhance the learning capability of model. Other CNN-based networks designed for medical image segmentation include nn-Unet (Isensee et al., 2018), Attention-Unet (Oktay et al., 2018), and ResUnet++ (Jha et al., 2019). Although CNNs excel at capturing local features due to their reliance on local receptive fields, this local focus limits their ability to model long-range dependencies. Convolution operations primarily focus on neighboring pixels, so that it is difficult for CNNs to effectively capture contextual information far from the target regions in the image. This lack of long-range dependency modeling hinders the network’s ability to fully understand global information, which can negatively affect segmentation or classification outcomes in polyp segmentation tasks.

Since the introduction of Transformer technology (Vaswani, 2017) into computer vision, it has effectively overcome the limitations of convolutional neural networks (CNNs) in capturing long-range dependencies. Vision Transformer (ViT) (Alexey, 2020) and Swin Transformer (Liu et al., 2021), widely used as backbone networks in vision tasks, offer a solid framework for modeling such dependencies. ViT relies on self-attention mechanisms, while Swin Transformer employs windowed self-attention and shifted windows to achieve similar results. Furthermore, Li et al. (2022) introduced the Contextual Transformer, which improves upon self-attention by incorporating neighborhood contextual information. However, traditional self-attention remains constrained by patch size, limiting token information to local regions and reducing the ability to capture global dependencies. In tasks like large polyp segmentation, a stronger global perception capability is necessary, as relying solely on patches as tokens is insufficient.

Currently, Transformer models are extensively used in medical image segmentation. Chen et al. (2021) proposed TransUnet, which merges the strengths of Transformer and U-Net, where the Transformer extracts global context and U-Net preserves local details, ensuring precise localization. Similarly, Cao et al. (2022) introduced Swin-Unet, which uses a hierarchical Swin Transformer encoder to extract contextual information via moving windows, coupled with a symmetric Swin Transformer decoder that restores spatial resolution using patch expansion layers. Moreover, Lin et al. (2022) developed Ds-TransUnet, which integrates the Swin Transformer into both the encoder and decoder of U-Net. Ds-TransUnet leverages a dual-scale encoding mechanism and an interaction fusion module, alongside the Transformer Interaction Fusion (TIF) module, to effectively combine multi-scale information through self-attention, facilitating non-local dependency modeling. Other Transformer-based medical segmentation algorithms include Transfuse (Zhang et al., 2021), UCTransNet (Wang et al., 2022), MT-UNet (Wang et al., 2022), and CoTr (Xie et al., 2021). However, due to the self-attention mechanism’s high computational complexity, Transformer models often struggle with convergence during training, particularly for long-sequence inputs, which hinders gradient propagation. Additionally, they lack an inherent inductive bias for local structures and require extensive data and training to learn effective features. These challenges result in slow and unstable convergence, especially in data-limited or resource-constrained environments.

Recently, State Space Model (SSM) methods, like Mamba (Gu and Dao, 2023), have demonstrated lower computational complexity in modeling long-range dependencies, allowing for faster convergence and offering a solution to the complexity issues associated with Transformer models. These methods have also been successfully applied to computer vision tasks. Zhu et al. (2024) introduced Vision Mamba, applying the Mamba model to image classification, while Ma et al. (2024) proposed U-Mamba, combining Mamba with U-Net for medical image segmentation. Liu et al. (2024) developed Swin-UMamba, blending the sliding window technique with the Mamba model to enhance segmentation accuracy. Xing et al. (2024) designed SegMamba, a 3D medical image segmentation model based on SSM, which excels in capturing long-range dependencies in volumetric data, offering greater efficiency than Transformers for high-resolution images. Although Mamba-based models reduce the computational burden of long-range dependency modeling, Transformers still outperform them in tasks requiring a longer context (Waleffe et al., 2024). In the case of polyp segmentation, distant micro-organism pixels may affect the final segmentation accuracy. Hence, enhancing the model’s ability to capture long-range dependencies while ensuring faster convergence is critical, alongside exploring the integration of strengths from both Transformer and Mamba models.

In summary, this study aims to enhance the algorithm’s ability to perceive long-range dependencies in polyp images, address the variations in polyp size and shape and ensure improved convergence speed. Consequently, VMDU-Net, a dual-encoder polyp segmentation network that integrates the strengths of both Transformer and Mamba models, is proposed. The contributions of this paper are as follows:

*Proposed VMDU-Net Model:* VMDU-Net, a dual-encoder multi-scale segmentation network, is introduced to tackle the challenges in polyp segmentation. Unlike previous dual-encoder algorithms, this model combines Transformer and Mamba architectures and incorporates Vision Mamba and Cross-Shape Transformer components. This significantly enhances the extraction of semantic information related to polyp shapes and boundaries, improves the model’s ability to capture long-range dependencies, and accelerates convergence.
*Design of the Cross-Shape Transformer:* A Cross-Shape Self-Attention mechanism is developed to replace the standard Self-Attention in traditional Transformers, resulting in the Cross-Shape Transformer. This mechanism utilizes cross-shaped regions as tokens and allows for more effective perception of long-range dependencies compared to patch-based Self-Attention.
*Design of the Mamba-Transformer-Merge:* The Mamba-Transformer-Merge module is introduced to effectively integrate features from both encoders. This module employs attention weighting across spatial and channel dimensions, maximizes the advantages of both Transformer and Mamba structures, and significantly enhances segmentation performance.

## 2 Related works

This part provides a thorough overview of the research advancements in colorectal polyp segmentation, encompassing a variety of methods ranging from traditional image processing approaches to the latest developments in machine learning and deep learning. Additionally, it emphasizes the historical development of these technologies. Special focus is placed on the role of Convolutional Neural Networks (CNNs) and Transformer models in boosting the accuracy and efficiency of segmentation. Through a systematic review of the progression of these techniques, this section outlines key technological innovations and methodological enhancements, offering readers a solid understanding of both the current trends and future directions in polyp segmentation.

### 2.1 Traditional algorithms for polyp segmentation

Traditional polyp segmentation methods can generally be divided into two categories: traditional image processing techniques and machine learning approaches. Traditional techniques include methods such as threshold-based segmentation, edge detection, and region-based segmentation, which focus on identifying features like color, texture, and shape in the images. In contrast, machine learning approaches are more effective at extracting color and texture features, particularly in polyp segmentation tasks. For instance, Guo et al. (2020) introduced a threshold model featuring a Threshold Map Supervised Generator (TMSG) that directs threshold learning to improve segmentation performance. Their dual-branch framework combines threshold learning with segmentation to enhance accuracy. Similarly, Ratheesh et al. (2016) presented an innovative polyp detection algorithm that improves accuracy by merging two segmentation techniques: the first uses linear thresholding to detect saturated regions in HSV images, while the second applies Markov Random Fields for deeper segmentation. This algorithm, designed to extract color and texture features from endoscopic images, stands out for its simplicity, speed, and effectiveness, providing reliable assistance to radiologists in detecting polyps.

Despite the progress achieved with these methods, traditional colorectal polyp segmentation still depends heavily on operator expertise and manual feature selection. This reliance on human knowledge introduces variability and often leads to subpar segmentation results. To meet the growing demands for accuracy and efficiency in real-world applications, developing more robust and automated segmentation methods is essential.

### 2.2 CNN for polyp segmentation

With the advent of Convolutional Neural Networks (CNNs), many CNN-based algorithms have been successfully adapted for general medical image segmentation and subsequently applied to polyp segmentation tasks. Prominent examples include U-Net by Ronneberger et al. (2015) and U-Net++ introduced by Zhou et al. (2018). Beyond these classic CNN models, some techniques have been developed specifically for polyp segmentation, such as ResU-Net by Diakogiannis et al. (2020) and ResU-Net++ by Jha et al. (2019). The ResU-Net family leverages residual learning to extract detailed micro-tissue and micro-structure features from polyp images, demonstrating strong segmentation performance. Additionally, Fan et al. (2020) proposed the PraNet algorithm, which enhances segmentation by aggregating high-level features through parallel sub-decoders and utilizing an inverse attention module to detect boundary cues, thereby improving the model’s ability to connect regions and boundaries. Banik et al. (2020) introduced Polyp-Net, a hybrid polyp segmentation network aimed at overcoming the limitations of traditional manual screening in colorectal cancer diagnosis. This model combines a Dual-Tree Wavelet Pooling Convolutional Neural Network (DT-WpCNN) with a Local Gradient Weighted Embedding Level Set Method (LGWe-LSM), which helps in extracting deep features, reducing false positives, and boosting segmentation accuracy. Sun et al. (2019) employed dilated convolutions to capture multi-scale high-level semantic features, simplifying the decoder’s feature fusion and reducing parameter count. Kim et al. (2021) introduced the Uncertainty-Aware Context Attention Network (UACANet), which enhances the model’s focus on polyp regions by leveraging uncertainty-aware attention mechanisms. Similarly, Zhang et al. (2020) presented an adaptive context selection encoding-decoding framework to address the challenges posed by the varying shapes and sizes of polyps. Furthermore, Yeung et al. (2021) developed Focus U-Net, a dual-attention-guided network that integrates spatial and channel attention into a Focus Gate module, improving the selective learning of polyp features.

While CNNs are highly effective at capturing local features due to their reliance on localized receptive fields, this very characteristic can limit their ability to capture long-range dependencies, restricting their use of global contextual information. As a result, CNNs may struggle to fully understand overall structures and intricate spatial relationships in polyp segmentation tasks, which can negatively impact model performance.

### 2.3 Transformer for polyp segmentation

Convolutional Neural Networks (CNNs) excel in polyp segmentation due to their strength in capturing local features. However, their ability to model global context and long-range dependencies is limited. In contrast, Transformers, with their self-attention mechanisms, are better equipped to capture global features and address CNNs’ shortcomings. As research on Transformers for image segmentation has progressed, numerous models have incorporated Transformer components to improve both the accuracy and robustness of polyp segmentation.

For example, TransFuse (Zhang et al., 2021) combines the strengths of both CNNs and Transformers, enabling the capture of global dependencies alongside low-level spatial details. The model uses a BiFusion module to efficiently merge multi-layer features from both architectures. Similarly, Duc et al. (2022) introduced ColonFormer, an encoder-decoder model that captures long-range semantic information across branches. Its encoder utilizes a lightweight Transformer to model global semantic relationships at multiple scales, thus improving polyp representation. Sanderson and Matuszewski (2022) developed the FCN-Transformer architecture, which combines Transformers with fully convolutional networks (FCNs). The main branch leverages the Transformer for feature extraction, while an auxiliary convolutional branch compensates for limitations in full-size prediction. Features from both branches are fused to generate a complete segmentation map. Additionally, Park and Lee (2022) proposed SwinE-Net, which combines EfficientNet, a CNN-based model, with the Swin Transformer. This integration, alongside multiple dilated convolution blocks, helps generate detailed feature maps, enhancing feature discriminability while retaining global semantic information and low-level CNN features. Dong et al. (2021) introduced Polyp-PVT, incorporating three core modules—Cascaded Fusion Module (CFM), Camouflage Identification Module (CIM), and Similarity Aggregation Module (SAM)—to address feature transfer and fusion limitations in traditional CNN-based models, thus achieving effective multi-level feature extraction. Meanwhile, Xiao et al. (2024) designed CTNet to handle challenges like polyp camouflage and size variability, employing long-range dependencies and structured feature maps for precise localization of camouflaged polyps. Other Transformer-based models for polyp segmentation include TransNetR by Jha et al. (2024), TransResU-Net by Tomar et al. (2022), and META-Unet by Wu et al. (2023), UCTNet by Guo et al. (2024), Multi-scale dual-channel feature embedding decoder method by Agarwal et al. (2024), Compound attention embedded dual channel encoder-decoder method by Ghosal et al. (2024).

Despite their advantages, Transformer-based models face challenges like slower convergence rates and higher computational complexity. The self-attention mechanisms used by Transformers, while powerful, are computationally intensive, leading to increased time complexity when processing long sequences. Additionally, selecting an appropriate learning rate can be difficult. A low rate may cause slow convergence, whereas a high rate can destabilize training. Furthermore, Transformers are less effective at capturing local features, which can reduce convergence efficiency for certain tasks. Their performance is also highly sensitive to the quality and diversity of training data—insufficient or highly variable data can further prolong the convergence process.

### 2.4 Mamba for polyp segmentation

In comparison to Transformer models, the Mamba model achieves long-range dependency capabilities with faster convergence rates. U-Mamba (Ma et al., 2024) integrates the Mamba model with U-Net and enhances long-range dependency without increasing computational complexity. Ruan et al. (2024) introduced VM-UNet, which incorporates a Visual State Space (VSS) block as a fundamental module to capture extensive contextual information. Furthermore, Tang et al. (2024) proposed RM-UNet, which features a Residual Visual State Space (ResVSS) module and a Rotational State Space Model (SSM) module to mitigate the efficiency reduction when transferring information from shallow to deep layers. The Rotational SSM module addresses the challenges of channel feature extraction within state space models. Fan et al. (2024) presented the SliceMamba model, which includes an efficient Bidirectional Slicing Scan (BSS) module that performs bidirectional feature slicing and applies different scanning mechanisms for slices with varying shapes. This design ensures the spatially adjacent features to remain close during the scanning sequence, so that segmentation performance is enhanced. Haggar and Boushey (2009) introduced HC-Mamba, which combines the Mamba model with convolutions for polyp segmentation, effectively captures long-range dependencies and maintains local information perception. Zhang et al. (2024) proposed HMT-UNet, which fuses the Mamba model with the Transformer model in the segmentation network.

Although the Mamba model effectively captures long-range dependencies and reduces computational complexity, its ability to do so is still inferior to that of Transformers.

### 2.5 Analysis of previous work

In spite of some technological advancements in colorectal polyp segmentation research, there are still lots of challenges. Traditional image processing methods, such as threshold segmentation and edge detection, rely heavily on the expertise and manual feature selection of operator, which can lead to inaccuracies and increased uncertainty. Therefore, more automated and stable segmentation techniques should be developed to enhance efficiency and precision.

Although Convolutional Neural Networks (CNNs) excel at local feature extraction, their limited receptive fields restrict their ability to model long-range dependencies and influence the understanding of overall image structure. In contrast, the Transformer architecture effectively captures global features. However, its computational complexity and slow convergence due to the self-attention mechanism hinder its application in medical image segmentation. Furthermore, Transformers have a relatively weak ability to extract local features, particularly when processing diverse medical image data, which is constrained by the quality and diversity of the training data.

Although the Mamba model converges faster than Transformers, it still falls short in modeling long-range dependencies and does not completely overcome the limitations of traditional methods. Thus, approaches that combine multi-scale feature extraction with attention mechanisms are a crucial research direction for improving the accuracy and robustness of polyp segmentation.

## 3 Method

This section introduces the proposed polyp segmentation network, VMDU-Net, along with its components, providing a detailed description of each component.

### 3.1 Overall architecture

**Figure 2:**
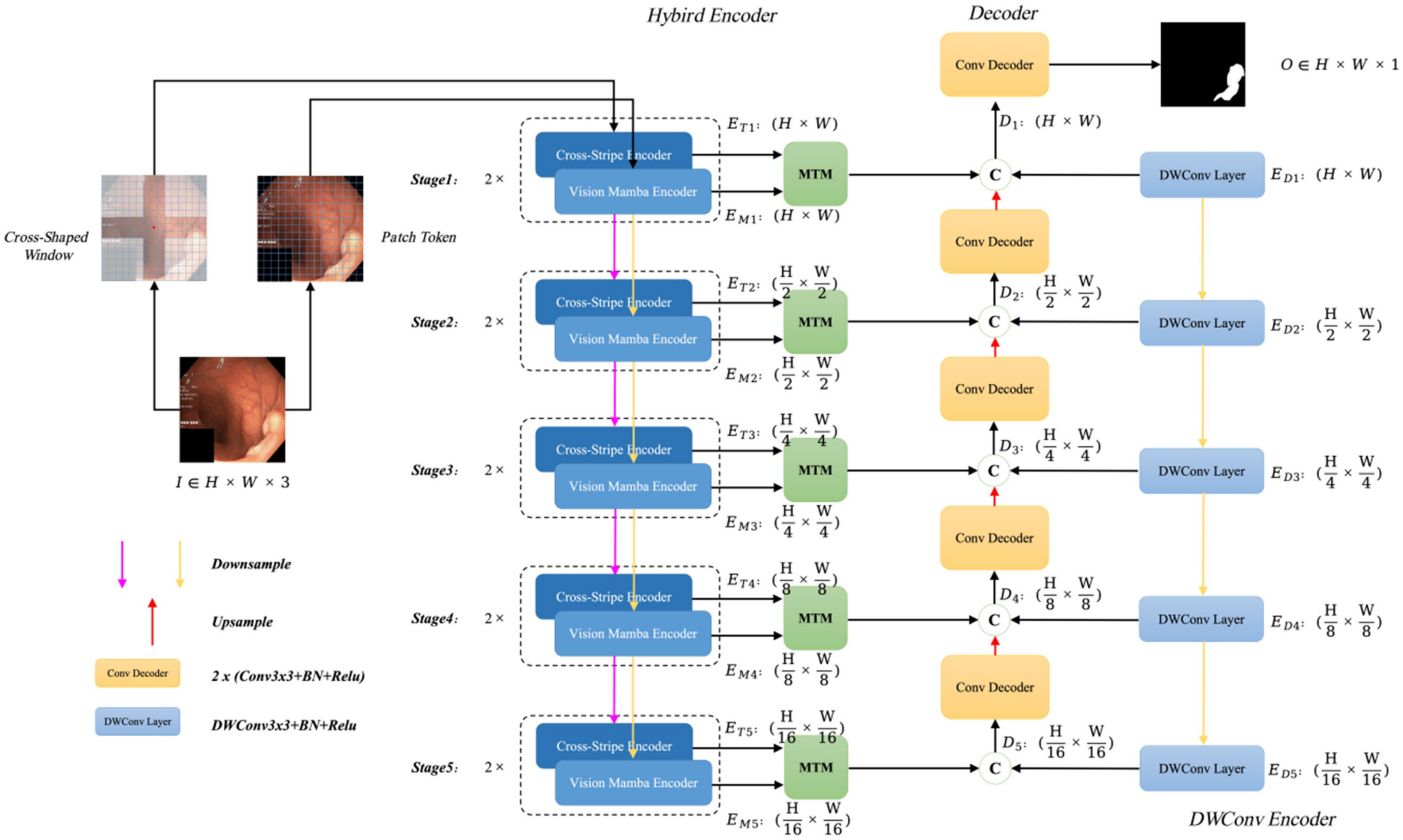
The overall architecture of VMDU-Net, including a dual encoder consisting of the Cross Shape Transformer and Vision Mamba for feature extraction, as well as the Mamba-Transformer-Merge (MTM) for merging features from the Transformer and Mamba.

The polyp segmentation network proposed in this paper, VMDU-Net, is illustrated in Figure 2.

VMDU-Net employs a dual-encoder design and incorporates a Cross Shape Self-Attention (CSA) mechanism based on a cross-window shape as tokens in one branch. The CSA is utilized to construct the Cross Shape Transformer (CST), which serves as the core structure of the first encoder. The second encoder integrates the Vision Mamba Encoder (VME) from Vision Mamba (Zhu et al., 2024) as its main component. Both encoders leverage a multi-scale feature extraction approach divided into five stages, in which each stage is downsampled to half the size of the previous stage through bilinear interpolation. At each stage, the features extracted by CST and VME are merged via the Mamba-Transformer-Merge (MTM) module. In addition to the dual-branch encoders, each level incorporates a lightweight Depthwise Separable Convolution layer as an auxiliary layer to provide bias-inducing information for both the Transformer and Mamba components. During the decoder phase, each decoder consists of standard convolution layers that receive three feature inputs: the output features from the MTM module, the output features from the previous decoder layer, and the output features from the Depthwise Separable Convolution layer.

Assuming the input image is denoted as $I\in {\mathbb{R}}^{H\times W\times 3}$ , at the i -th stage, the feature map output by CST has a size of $E_{\mathrm{Ti}}\in {\mathbb{R}}^{\frac{H}{2}\times \frac{W}{2}\times C_{i}}$ , the feature map output by VME has a size of $E_{\mathrm{Mi}}\in {\mathbb{R}}^{\frac{H}{2}\times \frac{W}{2}\times C_{i}}$ , the output feature size from the Depthwise Separable Convolution layer is $E_{\mathrm{Di}}\in {\mathbb{R}}^{\frac{H}{2}\times \frac{W}{2}\times C_{i}}$ , and the output size of each decoder layer is $D_{i}\in {\mathbb{R}}^{\frac{H}{2}\times \frac{W}{2}\times C_{i}}$ , $C_{i}\in \{32,64,128,256,512\}$ . Thus, the resulting segmentation map after processing through the VMDU-Net is denoted as $O\in {\mathbb{R}}^{H\times W\times 3}$ .

In the encoder section, the Mamba model has fewer parameters and lower computational cost, allowing for faster convergence, while the Transformer converges more slowly. Therefore, Mamba plays a critical role in capturing long-range dependencies during the early stages of training. As training progresses, the Transformer further explores deeper long-range dependencies to enhance the model’s semantic perception capabilities.

### 3.2 Cross Shape Transformer encoder

**Figure 3:**
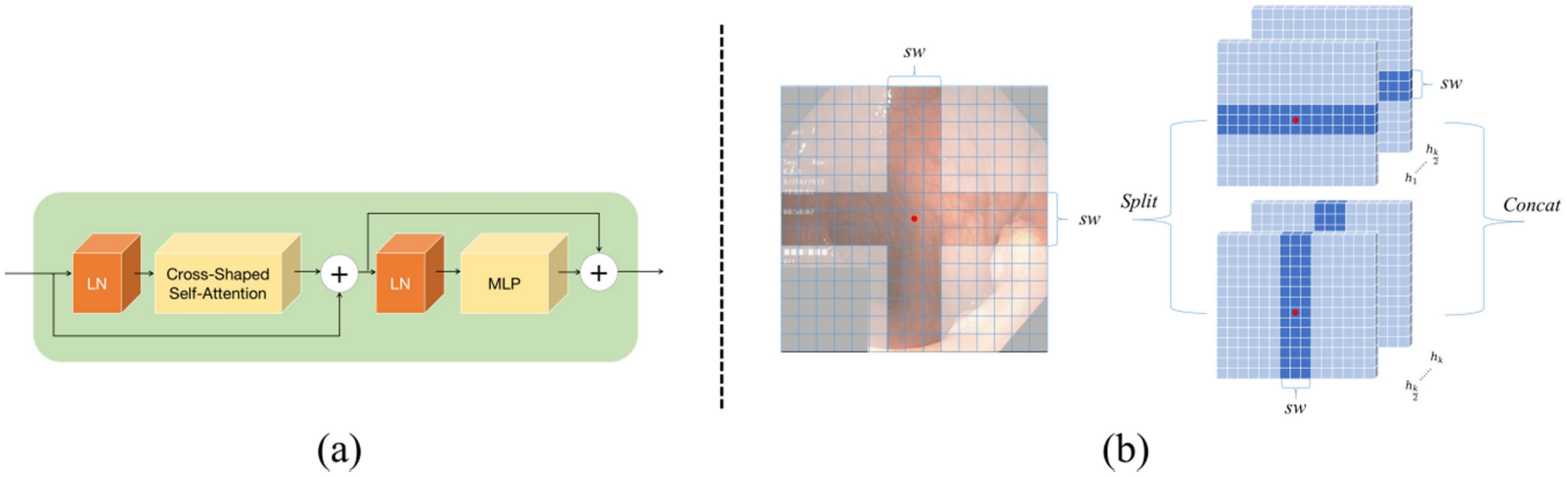
Cross Shape Transformer network structure: **(a)** shows the components of the Cross Shape Transformer, and **(b)** illustrates the cross-shaped region in the Cross Shape Self-Attention.

In order to enable the network structure to capture strong long-range dependencies, this study has designed the Cross Shape Transformer (CST) as the core component of the first encoder, as illustrated in Figure 3a. The CST consists of layer normalization, Cross Shape Self-Attention (CSA), and a multi-layer perceptron (MLP). In this architecture, each layer normalization module is equipped with residual connections to effectively mitigate the gradient vanishing problem during training. The MLP includes two layers and employs the GELU activation function to enhance nonlinear expressive capabilities of the model. The computational process of this Transformer can be represented as follows:

(1) $${\hat{X}}_{l}=\mathrm{CSA}(\mathrm{LN}(X_{l-1}))+X_{l-1}$$

(2) $$X_{l}=\mathrm{MLP}(\mathrm{LN}({\hat{X}}_{l}))+{\hat{X}}_{l}$$

In Equations 1, 2, the output from the previous layer is represented, while the current layer’s output is denoted. The feature maps are fed into the long-range dependency perception module within this structure. In the design of the Cross Shape Transformer, a specialized cross-shaped window is constructed, which allows parallel self-attention calculations along its horizontal and vertical strips, and thus implements Cross Shape Self-Attention (CSA), as shown in Figure 3b. This approach enables the model to effectively capture relationships between more distant pixels in the image, so that its overall feature extraction capabilities are enhanced.

In the CSA, the input features are first linearly projected into K heads. Subsequently, each head performs local self-attention calculations on the horizontal or vertical strips. During the self-attention computation on the horizontal strips, the feature X is evenly divided into multiple non-overlapping horizontal strips $\left[X^{1},X^{2},\ldots ,X^{M}\right]$ , each containing sw×W tokens, where sw represents the width of the strips. This width can be adjusted as needed to balance learning capacity and computational complexity. Formally, let $W_{k}^{Q}$ , $W_{k}^{K}$ , $W_{k}^{V}$ denote the projection dimensions for the query, key, and value of the k -th head, respectively. The output of the k -th head after performing self-attention calculations on the horizontal strips can be expressed as follows:

(3) $$X=[X^{1},X^{2},\ldots ,X^{M}]$$

(4) $$Y_{k}^{i}=\mathrm{Att}(X^{1}W_{k}^{Q},X^{2}W_{k}^{K},X^{M}W_{k}^{V})$$

(5) $${\text{HAtt}}_{k}(X)=[Y_{k}^{1},Y_{k}^{2},\ldots ,Y_{k}^{M}]$$

In Equations 3, 4, and 5, $X^{i}\in {\mathbb{R}}^{(\mathrm{sw}\times W)\times C}$ , M∈H/sw , i=1,…, M, and $W_{k}^{Q}\in {\mathbb{R}}^{C\times d_{k}}$ , $W_{k}^{K}\in {\mathbb{R}}^{C\times d_{k}}$ , $W_{k}^{V}\in {\mathbb{R}}^{C\times d_{k}}$ represent the query, key, and value projection matrices used by the k -th head, respectively. The term C/K represents how the feature dimensions are divided. For the self-attention computation on the vertical strips, a similar derivation is applied, with the output for the k -th head represented as ${\text{VAtt}}_{k}(X)$ . In view of the characteristics of head and neck medical images, this study assumes no directional bias. In this study, all K heads are divided into two groups, each containing K/2 heads, with K typically being an even number. The first group is in charge of performing self-attention computations on the horizontal strips, while the second group focuses on the vertical strips. Finally, the outputs from both groups are concatenated to form the complete feature representation.

(6) $$\mathrm{CSA}(X)=\text{Concat}({\text{head}}_{1},\ldots ,{\text{head}}_{k})W^{O}$$

(7) $${\text{head}}_{k}=\{\begin{array}{c} {\text{HAtt}}_{k}(X),k=1,\ldots ,\frac{K}{2}\\ {\text{VAtt}}_{k}(X),k=\frac{K}{2},\ldots ,K \end{array}$$

In Equations 6, 7, CSA represents Cross-Shape Self-Attention, while $W^{O}$ is the standard projection matrix used to convert the self-attention output into the target output dimension, typically set to C. As mentioned earlier, a key concept in the design of this self-attention mechanism is dividing the multi-head attention into several groups, each employing different self-attention operations. This grouping approach expands the attention field of each token within the Transformer module. This is contrasted with traditional self-attention mechanisms (Vaswani, 2017), which apply a uniform self-attention calculation across all heads.

### 3.3 Vision Mamba Encoder

**Figure 4:**
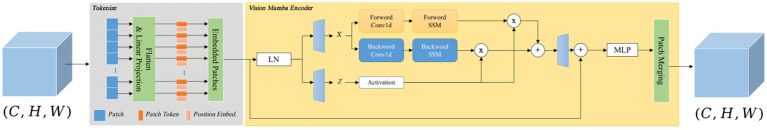
Vision Mamba Encoder structure consists of two parts: one part is Tokenlize, which is charge of tokenizing the input image into patches, and the other part is the Vision Mamba Encoder, which captures long-range dependencies. Finally, Patch Merging is adopted to convert the vectors into feature maps.

As shown in Figure 4, the Vision Mamba Encoder and Vision Transformer Encoder share a similar architecture. Given that the original Mamba encoder is primarily designed for processing 1D sequences, it is essential to modify the visual tasks. Specifically, the input 2D image is first divided into small patches and flattened. Let $t\in {\mathbb{R}}^{H\times W\times C}$ represent the image patch, with each patch containing C channels and a size of $x_{p}\in {\mathbb{R}}^{J\times (p^{2}C)}$ . Here, P represents the Patch Size, and $x_{p}$ is linearly projected into a vector of size D. Position encoding $E_{\mathrm{pos}}\in {\mathbb{R}}^{(J+1)\times D}$ is then added to these flattened patches, which are linearly projected into feature vectors of dimension D, with positional embedding incorporated. The entire process can be described as follows:

(8) $$T_{0}=[t_{\mathrm{cls}};t_{p}^{1}W;t_{p}^{2}W;\ldots ;t_{p}^{J}W;]+E_{\mathrm{pos}}$$

In Equation 8, $t_{p}^{J}$ represents the J-th patch of the image, while $W\in {\mathbb{R}}^{(p^{2}C)\times D}$ denotes the projection matrix. The sequence $T_{l-1}$ is then passed through the l-th layer of the Vision Mamba Encoder to generate the output $T_{l}$ . Finally, the class token $t_{0}^{L}$ , after normalization, is fed into a multi-layer perceptron (MLP) head, and produces the final prediction P. The detailed process is as follows:

(9) $$T_{l}=\mathrm{Vim}(T_{l-1})+T_{l-1}$$

(10) $$f=\text{Norm}(t_{0}^{L})$$

(11) p=MLP(f)

In Equations 9–11, VME refers to the Vision Mamba Encoder, Norm denotes the normalization layer, and MLP represents the multi-layer perceptron.

Due to the fact that the traditional Mamba module is primarily designed for one-dimensional sequences, it struggles to effectively manage spatial information in visual tasks. To address this, the Vision Mamba Encoder incorporates a bidirectional sequence modeling strategy specifically optimized for visual data. In Algorithm 1, the Vision Mamba Encoder processes both forward and backward sequences, where each direction can employ distinct state-space parameters. This approach allows the model to simultaneously attend to the beginning and end of the sequence, capturing both spatial and contextual details more effectively. Before sequence processing begins, the input data is standardized using layer normalization (LN) to stabilize the training process and enhance overall performance. Following this, the normalized sequence is linearly projected into two independent spaces, which are subsequently used for bidirectional processing and gating via separate linear layers. Afterward, each direction independently processes the sequence, using 1D convolutions to capture local dependencies and produce the output, x1. This output is then passed through three linear layers to compute three critical parameters: B , C , and Δ . Additionally, parameter D undergoes a softplus transformation to ensure it remains positive, as it is integral to the temporal scaling transformation. The modified Δ serves as a scaling factor for the evolution matrix B and the input matrix C , with Δ regulating the scaling of these matrices. After this transformation, the state-space model computes the final output. The forward and backward outputs are then combined using a gating mechanism, multiplied spatially, and summed to generate the final sequence output. This process involves linear layers and residual connections, ultimately constructing the final sequence. Finally, the Patch Merging module reconstructs the output into $t\in {\mathbb{R}}^{H\times W\times C}$ .

**ALGORITHM 1:**
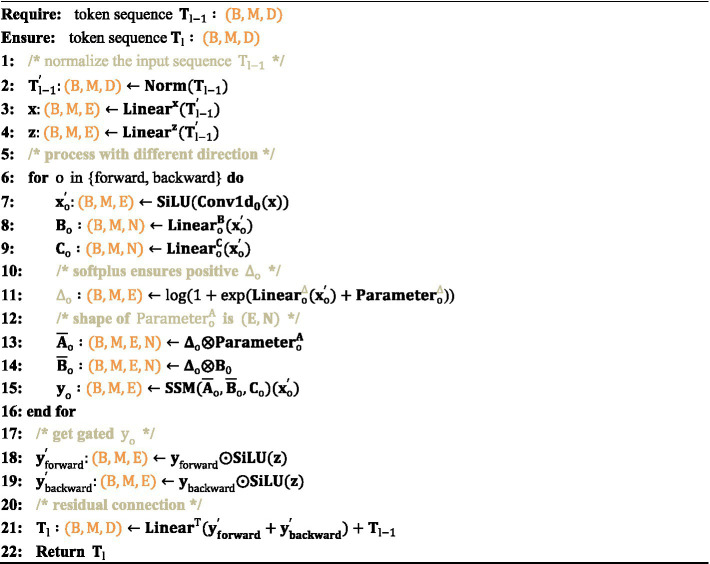
Vision Mamba Encoder process.

### 3.4 Mamba-Transformer-Merge

**Figure 5:**
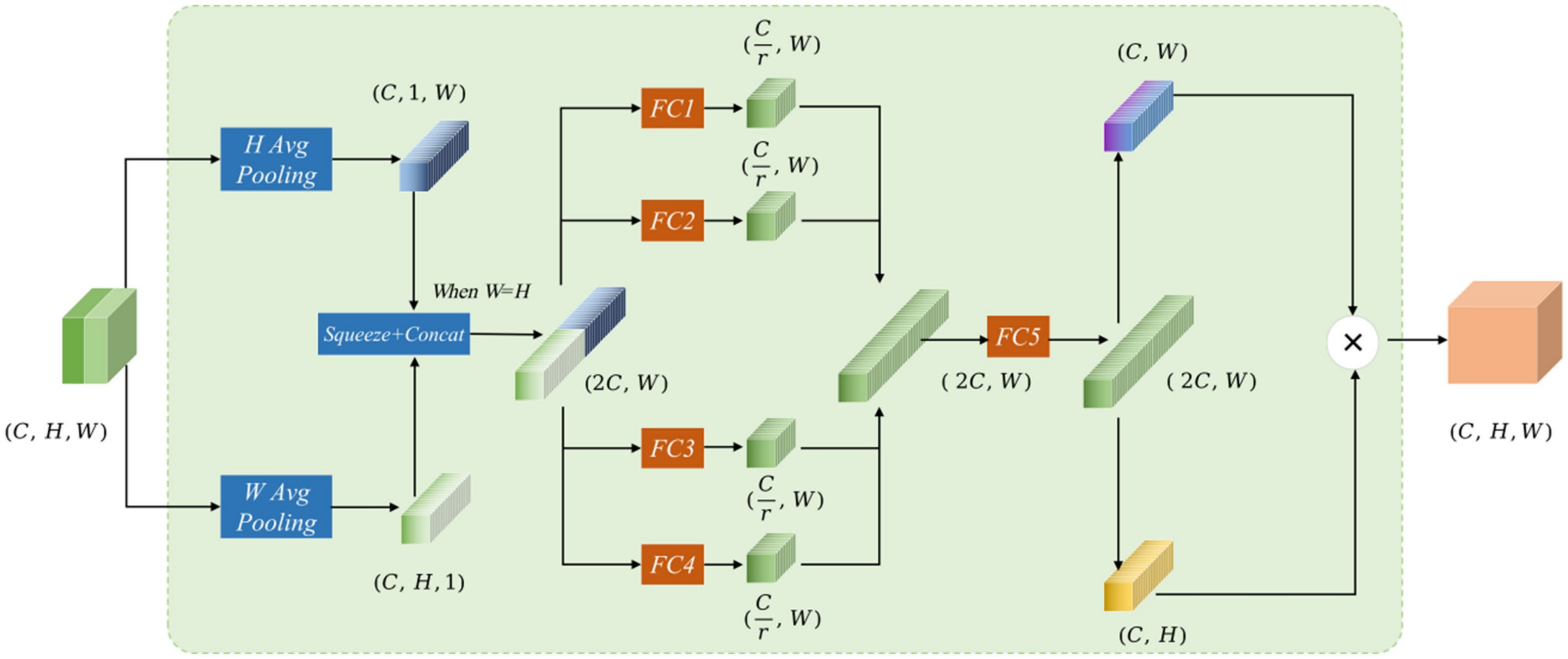
The Mamba-Transformer-Merge network structure features red and green feature maps in the input, representing the concatenation of outputs from the Cross Shape Transformer and the Vision Mamba Encoder, respectively. The input and output dimensions of the MTM module are the same.

The Mamba-Transformer-Merge (MTM) module’s network structure combines feature maps obtained from the Cross Shape Transformer and the Vision Mamba Encoder at each stage. In the input representation, the red feature map signifies the output from the Cross Shape Transformer, whereas the green feature map represents the output from the Vision Mamba Encoder. Subsequently, the MTM module concatenates and fuses these two feature maps (see Figure 5).

Assuming the input feature of the MTM module is $X\in {\mathbb{R}}^{H\times W\times C}$ , the processing occurs through several steps. To improve long-range interactions and accurately capture spatial information, local attention is split into two branches: one that performs pooling in the H-direction and another that does so in the W-direction. The resulting vectors decompose the original feature map into horizontal and vertical components, with each coordinate encoding distinct pixel information. For the input X, the output in the H-direction across the C channels can be expressed as:

(12) $$Z_{c}^{h}(h)=\frac{1}{W}\sum_{0\leq m<W}X_{c}(h,m)$$

Output in the W-direction across the C channels can be expressed as:

(13) $$Z_{c}^{w}(w)=\frac{1}{H}\sum_{0\leq m<H}X_{c}(w,m)$$

In Equations 12, 13, the two feature maps are compressed into feature vectors of size $Z_{h}\in {\mathbb{R}}^{W\times C}$ and $Z_{w}\in {\mathbb{R}}^{H\times C}$ , respectively. When H = W, these two feature maps are concatenated to form a feature vector of size $Z\in {\mathbb{R}}^{W\times 2C}$ . Then, four fully connected branches are applied to reduce the dimensions of the concatenated vector to 1/4 of the input channels, producing four feature maps $f_{i}\in \{f_{1},f_{2},f_{3},f_{4}\}$ . After the four fully connected branches are formed, the resulting feature maps are concatenated to obtain a feature map of size $Z_{o}\in {\mathbb{R}}^{W\times 2C}$ .

(14) $$Z_{o}\in {\mathbb{R}}^{W\times 2C}=\text{Concat}(f_{1},f_{2},f_{3},f_{4})$$

In Equation 14, this is then split into feature vectors $O_{h}\in {\mathbb{R}}^{W\times C}$ and $O_{w}\in {\mathbb{R}}^{H\times C}$ , and matrix multiplication is performed on the two vectors to generate $O\in {\mathbb{R}}^{H\times W\times C}$ .

(15) $$O=O_{h}\times O_{w}$$

In Equation 15, the decomposition along the W and H directions is inspired by the coordinate attention mechanism, allowing for spatial position weighting during the attention calculation. The use of four fully connected branches for dimensionality reduction and concatenation follows the concept of channel attention weighting. Drawing from SENetV2 (Narayanan, 2023), multiple branches facilitate the perception of feature information from different dimensions.

## 4 Experiments and results

In this paper, the polyp segmentation network VMDU-Net is proposed. To evaluate its performance fairly, the study assesses VMDU-Net through five publicly available polyp datasets. This evaluation includes comparisons with classical algorithms, recent state-of-the-art (SOTA) methods, ablation studies, and visualization comparisons. In this section, these aspects will be elaborated.

### 4.1 Datasets

This study utilizes five publicly available datasets: Kvasir-SEG (Jha et al., 2019), CVC-ClinicDB (Silva et al., 2014), EndoScene (Bernal et al., 2015), CVC-ColonDB (Tajbakhsh et al., 2015), and ETIS (Vázquez et al., 2017), which are widely used to evaluate most of the current polyp segmentation models, such as Polyp-PVT (Dong et al., 2021) and DEMF-Net (Cao et al., 2024).

*Kvasir-SEG*: The Kvasir-SEG dataset consists of 1,000 polyp images with ground truth annotations. The image resolutions vary from 332 × 487 to 1,920 × 1,072 pixels, offering a wide range of image quality and detail.
*CVC-ClinicDB*: CVC-ClinicDB is an open-access dataset comprising 612 images extracted from 31 colonoscopy sequences, each with a resolution of 384 × 288 pixels. This dataset is mainly used for medical image segmentation, especially for polyp detection in colonoscopy videos.
*EndoScene*: EndoScene provides 912 annotated images created by merging CVC-ClinicDB and CVC300 datasets, offering a richer variety of samples for polyp segmentation research.
*CVC-ColonDB*: This dataset is based on 15 different colonoscopy sequences and contains 380 polyp images, all standardized to 574 × 500 pixels with corresponding annotations.
*ETIS*: The ETIS dataset contains 192 polyp images and their annotations from 29 colonoscopy sequences, with each image uniformly sized at 1,225 × 996 pixels, ensuring consistency across the data.

During training, we combine the training sets of Kvasir-SEG and CVC-ClinicDB to create a new dataset for training the VMDU-Net model. For testing, the test sets from Kvasir-SEG and CVC-ClinicDB are used as in-distribution data, while EndoScene, CVC-ColonDB, and ETIS—datasets not involved in training—serve as out-of-distribution test data. This approach allows for a comprehensive evaluation of the model’s generalization performance across different data distributions.

### 4.2 Implementation details

In this study, the VMDU-Net architecture is implemented alongside several comparative algorithms, including U-Net. The implementation uses PyTorch 1.10. To ensure an objective evaluation of VMDU-Net, all reproduced PyTorch network architectures are integrated into the MMsegmentation framework. This integration ensures consistency in input and output dimensions, preprocessing techniques, training epochs, loss functions, and metric calculations. To maintain fairness, the study does not utilize pretrained weights for any of the networks during training. Training occurs on four Quadro RTX 8000 GPUs, each equipped with 48GB of memory.

Various hyperparameters and data preprocessing strategies are employed throughout the experiments. Input images are resized to (384, 384) pixels and normalized to have a mean of 0 and a standard deviation of 1. Data augmentation techniques include random flipping, photometric distortion, padding, and random warping. The optimizer used is Adam, with a learning rate of 1e-4, and a polynomial learning rate schedule is applied with an exponent of 0.9. The model trains for 5,000 iterations with a batch size of 8.

The performance of the model is assessed using four key metrics: mIoU, Dice, Precision, and Recall. Mean Intersection over Union (mIoU) serves as a widely used metric for evaluating model accuracy in semantic segmentation tasks; it computes the ratio of the intersection to the union of the predicted and ground truth areas, then averages these results. The Dice coefficient, which ranges from 0 to 1, quantifies overlap, with values approaching 1 indicating a higher degree of similarity in segmentation outcomes. Precision measures the fraction of true positive samples among all samples predicted as positive, reflecting the model’s accuracy. Recall assesses the proportion of correctly predicted positive samples among all actual positive cases, representing the model’s sensitivity. Collectively, these four metrics provide a comprehensive evaluation of model performance.

In terms of the loss function, polyp segmentation is considered as a binary classification problem. Thus, Binary Cross-Entropy (BCE) loss is leveraged. Given that the gradient flow information at polyp boundaries is rich, Dice loss is also used to enhance the accuracy of positive and negative sample predictions. The total loss $L_{\text{Total}}$ is expressed as follows:


(16) $$L_{\text{Total}}=\alpha L_{\mathrm{Bce}}(O,G)+\beta L_{\text{Dice}}(O,G)$$

In Equation 16, let O represent the segmentation map predicted by the network and G denote the ground truth labels. The BCE loss is denoted as $L_{\mathrm{Bce}}$ and the Dice loss as $L_{\text{Dice}}$ . The parameters α and β represent the weights for each loss component. In the experiments, this study validated that the optimal accuracy is achieved when α = β =1.

### 4.3 Comparative experiments

In the comparative experiments, this study implemented the VMDU-Net architecture and reproduced several benchmark algorithms, including U-Net (Ronneberger et al., 2015) and PraNet (Fan et al., 2020), which are convolutional neural network-based medical image segmentation methods. At the same time, Transformer-based algorithms such as TransUnet (Chen et al., 2021) and SwinUnet (Cao et al., 2022) were included. Additionally, Mamba model based U-Mamba (Ma et al., 2024) and VM-Unet (Guo et al., 2024) were selected. Algorithms such as Focus-Unet (Yeung et al., 2021) and Polyp-PVT (Dong et al., 2021) represent recent state-of-the-art (SOTA) methods in polyp segmentation. These algorithms were employed for an objective evaluation of VMDU-Unet, with results presented in Tables 1, 2.

**Table 1:** The comparison experiment results on the Kvasir-SEG dataset and CVC-ClinicDB use four metrics: Dice, mIoU, Precision, and Recall.

Type	Model	Kvasir-SEG				CVC-ClinicDB			
		Dice	mIoU	Precision	Recall	Dice	mIoU	Precision	Recall
CNN	U-Net (Ronneberger et al., 2015)	0.812	0.721	0.853	0.831	0.834	0.751	0.882	0.857
	U-Net++ (Zhou et al., 2018)	0.723	0.637	0.832	0.764	0.716	0.607	0.819	0.771
	ResUnet (Diakogiannis et al., 2020)	0.835	0.745	0.865	0.815	0.814	0.785	0.854	0.793
	ResUnet++ (Jha et al., 2019)	0.811	0.792	0.878	0.703	0.799	0.791	0.879	0.705
	PraNet (Fan et al., 2020)	0.897	0.844	0.907	0.914	0.898	0.843	0.963	0.913
	Focus U-Net (Yeung et al., 2021)	0.911	0.847	0.913	0.915	0.942	0.895	0.953	0.933
Transformer	TransUnet (Chen et al., 2021)	0.917	0.951	0.936	0.892	0.938	0.889	0.927	0.926
	SwinUnet (Cao et al., 2022)	0.915	0.864	0.928	0.912	0.914	0.877	0.929	0.889
	SwinE-Net (Park and Lee, 2022)	0.926	0.862	0.924	0.928	0.925	0.914	0.922	0.919
	Polyp-PVT (Dong et al., 2021)	0.918	0.868	0.913	0.896	0.933	0.887	0.935	0.911
	DEMF-Net (Cao et al., 2024)	0.913	0.865	0.911	0.935	0.958	0.917	0.965	0.951
Mamba	U-Mamba (Gu and Dao, 2023)	0.906	0.857	0.913	0.918	0.926	0.905	0.932	0.918
	VM-UNet (Ruan et al., 2024)	0.912	0.852	0.922	0.913	0.938	0.904	0.941	0.913
	Polpy-Mamba (Zhu et al., 2025)	0.915	0.853	0.925	0.914	0.940	0.911	0.944	0.920
	**VMDU-Net**	**0.938**	**0.871**	**0.933**	**0.938**	**0.964**	**0.932**	**0.971**	**0.959**

The bold values represent the highest metrics achieved by the algorithms used in this study.

**Table 2:** The comparison experiment results on the CVC-ColonDB dataset and ETIS and EndoScence datasets use two metrics: Dice, mIoU.

Type	Model	CVC-ColonDB		ETIS		EndoScence	
		Dice	mIoU	Dice	mIoU	Dice	mIoU
CNN	U-Net (Ronneberger et al., 2015)	0.812	0.721	0.401	0.340	0.627	0.535
	U-Net++ (Zhou et al., 2018)	0.723	0.637	0.297	0.247	0.428	0.357
	ResUnet (Diakogiannis et al., 2020)	0.835	0.745	0.152	0.089	0.591	0.511
	ResUnet++ (Jha et al., 2019)	0.811	0.792	0.121	0.081	0.834	0.777
	PraNet (Fan et al., 2020)	0.897	0.844	0.628	0.567	0.871	0.791
	Focus U-Net (Yeung et al., 2021)	0.911	0.847	0.590	0.528	0.760	0.688
Transformer	TransUnet (Chen et al., 2021)	0.917	0.951	0.593	0.551	0.754	0.682
	SwinUnet (Cao et al., 2022)	0.915	0.864	0.570	0.530	0.745	0.686
	SwinE-Net (Park and Lee, 2022)	0.926	0.862	0.590	0.508	0.762	0.695
	Polyp-PVT (Dong et al., 2021)	0.918	0.868	0.670	0.590	0.787	0.708
	DEMF-Net (Cao et al., 2024)	0.913	0.865	0.680	0.603	0.908	0.882
Mamba	U-Mamba (Gu and Dao, 2023)	0.906	0.857	0.614	0.545	0.856	0.804
	VM-UNet (Ruan et al., 2024)	0.912	0.852	0.675	0.582	0.898	0.823
	Polpy-Mamba (Zhu et al., 2025)	0.913	0.866	0.666	0.601	0.899	0.825
	**VMDU-Net**	**0.938**	**0.871**	**0.715**	**0.634**	**0.926**	**0.886**

The bold values represent the highest metrics achieved by the algorithms used in this study.

In Table 1, the results of the comparative experiments conducted on the Kvasir-SEG and CVC-ClinicDB datasets are presented. Due to the larger data volumes in Kvasir-SEG and CVC-ClinicDB among the five datasets, this study employed four evaluation metrics: Dice, mIoU, Precision, and Recall.

#### 4.3.1 Kvasir-SEG

In the CNN models, ResUnet and ResUnet++ showed improvements over U-Net. However, ResUnet++ had a Precision of 0.878 but a Recall of only 0.703, indicating an issue with insufficient recall. PraNet and Focus U-Net performed well among CNN models, with Focus U-Net achieving a Dice score of 0.911. Transformer-based models, such as TransUnet, SwinUnet, and SwinE-Net, significantly outperformed traditional CNNs in terms of Dice and mIoU, in which SwinE-Net achieved the best Dice score of 0.926. Polyp-PVT also demonstrated strong performance, highlighting the potential of Transformers in segmenting complex structures. U-Mamba and VM-UNet, based on the Mamba architecture, showed competitive results, but VMDU-Net achieved the overall best performance with a Dice score of 0.938 and a mIoU of 0.871. Overall, Transformer models significantly outperformed CNNs on the Kvasir-SEG dataset, with VMDU-Net achieving the best results, demonstrating a Dice coefficient of 0.938, a mIoU of 0.871, a Precision of 0.933, and a Recall of 0.938.

#### 4.3.2 CVC-ClinicDB

Within the CNN architectures, Focus U-Net and PraNet performed notably well, achieving Dice scores of 0.942 and 0.898, respectively, indicating strong segmentation capabilities. In contrast, U-Net++ performed poorly, with a Dice score of only 0.716, reflecting its limitations in handling complex structures. Transformer-based models, including DEMF-Net, TransUnet, and Polyp-PVT, also exhibited competitiveness. DEMF-Net achieved a Dice score of 0.958, a mIoU of 0.917, as well as Precision and Recall scores of 0.965 and 0.951, respectively, demonstrating strong segmentation accuracy and recall. VMDU-Net reached a Dice coefficient of 0.964, a mIoU of 0.932, and Precision and Recall scores of 0.971 and 0.959, respectively, showcasing an excellent balance between accuracy and recall. That is to say, the model accurately identifies most polyps, effectively captures additional polyp regions, and significantly enhances segmentation quality.

#### 4.3.3 CVC-ColonDB

In the CNN architectures, Focus U-Net and PraNet also performed well, achieving Dice scores of 0.911 and 0.897, respectively, with mIoU values exceeding 0.840, demonstrating their effectiveness in segmenting complex structures. In contrast, U-Net++ performed relatively poorly, with a Dice score of only 0.723, indicating its limitations in handling colorectal polyp segmentation tasks. Transformer-based models, such as TransUnet, SwinUnet, and Polyp-PVT, exhibited strong performance. Particularly, TransUnet demonstrated excellent segmentation results with a Dice score of 0.917 and an mIoU of 0.951. These models optimize the feature extraction process, enhancing their ability to capture fine details. On the CVC-ColonDB dataset, VMDU-Net maintained the best performanceand achieved a Dice coefficient of 0.938 as well as an mIoU of 0.871.

#### 4.3.4 ETIS

U-Net and U-Net++ showed weak performance, with Dice scores of only 0.401 and 0.297, reflecting their limitations on this dataset. ResUnet and ResUnet++ had even lower results, with Dice coefficients of 0.152 and 0.121, indicating significant shortcomings in extracting subtle structures. Among the better-performing models, DEMF-Net achieved a Dice score of 0.680 and a mIoU of 0.603. In addition, PraNet, Polyp-PVT, and VM-UNet also yielded relatively good results, with Dice scores of 0.628, 0.670, and 0.675, respectively. These models exhibited relatively strong segmentation capabilities, but overall performance remained below that of VMDU-Net, which achieved a Dice coefficient of 0.715 and a mIoU of 0.634.

#### 4.3.5 EndoScene

U-Net++ performed poorly, with a Dice score of only 0.428, reflecting its inadequacies in handling complex polyp structures. Even though ResUnet++ achieved a Dice score of 0.834, it still fell short compared to more advanced models. Among other models, DEMF-Net and VM-UNet also presented strong performance, achieving Dice scores of 0.908 and 0.898, respectively, indicating robust segmentation capabilities. PraNet attained a Dice score of 0.871, demonstrating its effectiveness in detail extraction. VMDU-Net excelled with a Dice coefficient of 0.926 and an mIoU of 0.886.

VMDU-Net achieved excellent results across all three datasets, and attained the highest scores in Dice and mIoU. It demonstrated strong capabilities in handling complex intestinal structures, and provided an effective solution for the automatic segmentation of colorectal polyps.

As shown in Table 3, we compare VMDU-Net with several existing algorithms in terms of parameter count and performance. The results show that VMDU-Net significantly reduces both computational cost and inference time compared to SOTA models such as Focus U-Net, Polyp-PVT, and DEMF-Net, achieving an impressive inference speed of 85.8 ms per image. However, it still falls short of pure Mamba-based architectures, mainly due to the additional computational overhead introduced by the dual-encoder design. Future work will focus on optimizing this structure to further improve efficiency.

**Table 3:** The performance comparison table is based on input images of size 256 × 256, tested on an RTX 3090 GPU, and reports the number of parameters, FLOPs, and inference time.

Method	FLOPs (GFLOPs)	Parameters (M)	Inference time (ms)
U-Net (Ronneberger et al., 2015)	29.8	31.5	18.2
U-Net++ (Zhou et al., 2018)	38.5	42.0	35.4
PraNet (Fan et al., 2020)	119.6	142.2	81.5
Focus U-Net (Yeung et al., 2021)	113.8	127.7	98.6
TransUnet (Chen et al., 2021)	162.4	105.3	139.3
SwinUnet (Cao et al., 2022)	123.5	62.0	90.0
Polyp-PVT (Dong et al., 2021)	245.9	227.7	221.7
DEMF-Net (Cao et al., 2024)	139.3	145.8	121.2
U-Mamba (Gu and Dao, 2023)	58.7	45.1	45.6
VM-UNet (Ruan et al., 2024)	61.2	44.3	41.3
VMDU-Net	135.1	102.2	85.8

### 4.4 Ablation experiment

In VMDU-Net, four components were utilized, including the Vision Mamba Encoder (VM), Cross Shape Transformer Encoder (CST), Mamba-Transformer-Merge (MTM), and Depthwise Separable Convolution Encoder (DWConv). To further investigate the contribution of each component, ablation experiments were conducted on the Kvasir-SEG and CVC-ClinicDB datasets. The results are shown in Tables 4, 5.

**Table 4:** The ablation experiment results on the Kvasir-SEG dataset evaluate four metrics: Dice, mIoU, Precision, and Recall.

Index	Setting				Kvasir-SEG			
	VM	CST	MTM	DWConv	Dice	mIoU	Precision	Recall
1	✓	×	×	×	0.893	0.832	0.898	0.901
2	×	✓	×	×	0.915	0.851	0.910	0.916
3	✓	×	×	✓	0.905	0.841	0.909	0.913
4	×	✓	×	✓	0.918	0.854	0.915	0.920
5	✓	✓	×	×	0.922	0.855	0.916	0.921
6	✓	✓	×	✓	0.924	0.860	0.918	0.923
7	✓	✓	✓	×	0.925	0.862	0.920	0.926
8	✓	✓	✓	✓	0.938	0.871	0.933	0.938

VM represents Vision Mamba Encoder, CST represents Cross Shape Transformer Encoder, MTM represents Mamba-Transformer-Merge, and DWConv represents Depthwise Separable Convolution Encoder. The bold values represent the highest metrics achieved by the algorithms used in this study.

**Table 5:** The ablation experiment results on the CVC-ClinicDB dataset evaluate four metrics: Dice, mIoU, Precision, and Recall.

Index	Setting				CVC-ClinicDB			
	VM	CST	MTM	DWConv	Dice	mIoU	Precision	Recall
1	✓	×	×	×	0.903	0.895	0.927	0.902
2	×	✓	×	×	0.917	0.857	0.912	0.915
3	✓	×	×	✓	0.914	0.852	0.908	0.911
4	×	✓	×	✓✓	0.926	0.860	0.918	0.920
5	✓	✓	×	×	0.931	0.871	0.924	0.925
6	✓	✓	×	✓	0.934	0.874	0.927	0.928
7	✓	✓	✓	×	0.958	0.925	0.965	0.952
8	✓	✓	✓	✓	**0.964**	**0.932**	**0.971**	**0.959**

VM represents Vision Mamba Encoder, CST represents Cross Shape Transformer Encoder, MTM represents Mamba-Transformer-Merge, and DWConv represents Depthwise Separable Convolution Encoder. The bold values represent the highest metrics achieved by the algorithms used in this study.

As indicated by the experimental results on the Kvasir-SEG dataset, when only the Vision Mamba Encoder (VM) is used, the model achieves a Dice score of 0.893 and a mIoU of 0.832, demonstrating its effectiveness in capturing long-range dependencies. However, with the addition of the Cross Shape Transformer Encoder (CST), performance significantly improves to a Dice score of 0.915 and an mIoU of 0.851, highlighting crucial role of CST in enhancing feature extraction capabilities. As revealed by further analysis, the combination of VM and CST leads to an even greater performance boost, with the Dice score reaching 0.922, indicating that the synergy between these two encoders facilitates a more comprehensive capture of image details. Besides, the introduction of the Mamba-Transformer-Merge (MTM) module enables more effective fusion of different feature maps, consistently enhancing performance across various experimental setups. Ultimately, when all components are activated, VMDU-Net achieves a Dice score of 0.938 and a mIoU of 0.871 on the Kvasir-SEG dataset, showcasing the effective collaboration of all modules. Overall, the ablation experiments not only validate the complementarity and necessity of the components in the VMDU-Net design, but also provide a robust performance foundation for polyp segmentation tasks.

In the ablation experiments on the CVC-ClinicDB dataset, the model achieved a Dice score of 0.903 and an mIoU of 0.895 when only the Vision Mamba Encoder (VM) was used, demonstrating its effectiveness in capturing long-range dependencies. However, with the introduction of the Cross Shape Transformer Encoder (CST), the Dice score improved to 0.917, despite slight decrease of the mIoU to 0.857, indicating CST’s exceptional performance in enhancing the semantic information extraction of features. Furthermore, when Depthwise Separable Convolution (DWConv) was combined with CST, the Dice score reached 0.926, signifying an enhancement in the model’s local information processing ability. When both VM and CST were employed, the model’s performance significantly improved, achieving a Dice score of 0.931 and a mIoU of 0.871. Ultimately, when all components were activated, VMDU-Net attained a Dice score of 0.964 and a mIoU of 0.932 on the CVC-ClinicDB dataset, demonstrating optimal performance. Evidently, the synergistic effect of all components greatly enhances the model’s segmentation capability for complex structures, validating the importance and complementarity of each module in the design.

The hyperparameter sw in the CSA module affects the model’s accuracy. In Table 6, we conduct an ablation study on sw in the CSA module. The model achieves the highest accuracy when sw = 3. A small sw makes it difficult to capture global information, while a large sw slows down convergence. Therefore, sw is set to 3.

**Table 6:** Ablation experiment of the hyperparameter sw in the CSA module, with sw set to 1, 3, 5, and 7.

sw	Kvasir-SEG				CVC-ClinicDB			
	Dice	mIoU	Precision	Recall	Dice	mIoU	Precision	Recall
1	0.920	0.850	0.915	0.915	0.950	0.910	0.960	0.950
3	0.938	0.871	0.933	0.938	0.964	0.932	0.971	0.959
5	0.932	0.864	0.927	0.933	0.960	0.924	0.965	0.953
7	0.928	0.860	0.923	0.927	0.957	0.919	0.962	0.950

**Figure 6:**
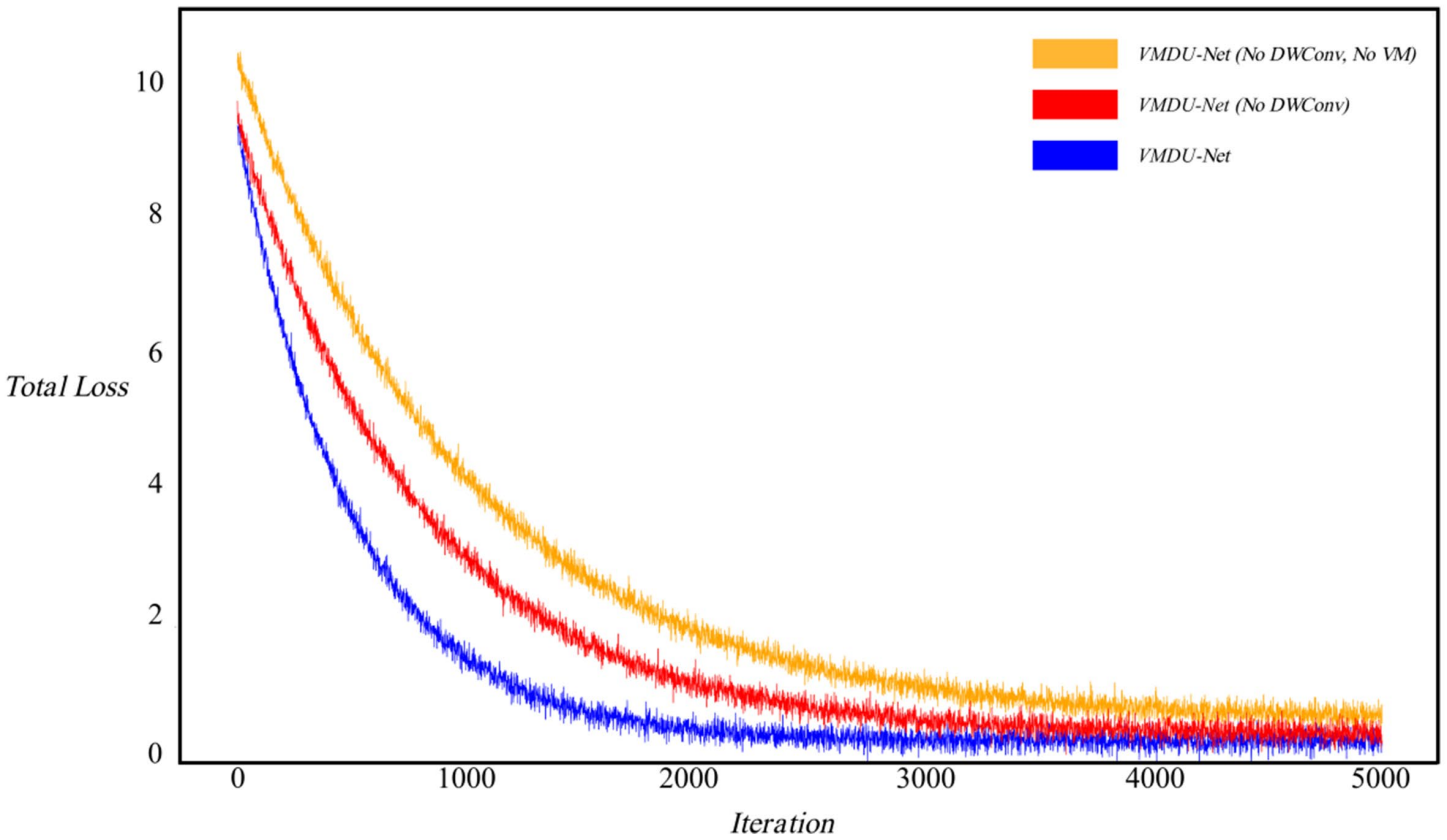
Ablation experiment loss curve, in which the horizontal axis represents iteration and the vertical axis represents Loss value. “No DWConv” means that Depthwise Separable Convolution is not used, and “No VM” means that the Vision Mamba Encoder is not used.

As shown in Figure 6, the loss curves were visualized to investigate the impact of the Vision Mamba Encoder (VM) and Depthwise Separable Convolution (DWConv) on model convergence. Without the use of DWConv and VM, the model exhibited the slowest convergence rate. After introduction of the Mamba model, the convergence speed improved, and the loss value significantly decreased. When prior feature information was provided through Depthwise Separable Convolution, the model achieved the fastest convergence rate.

### 4.5 Visualization

**Figure 7:**
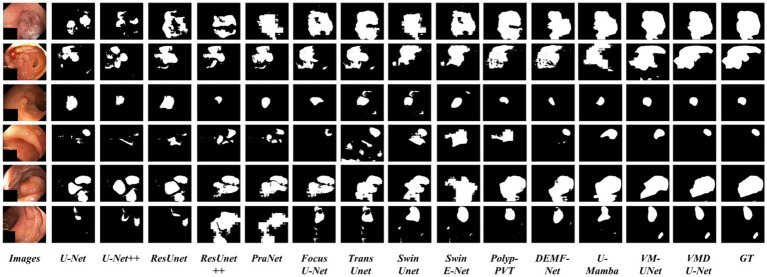
Partial visualization of segmentation results on the Kvasir-SEG dataset.

**Figure 8:**
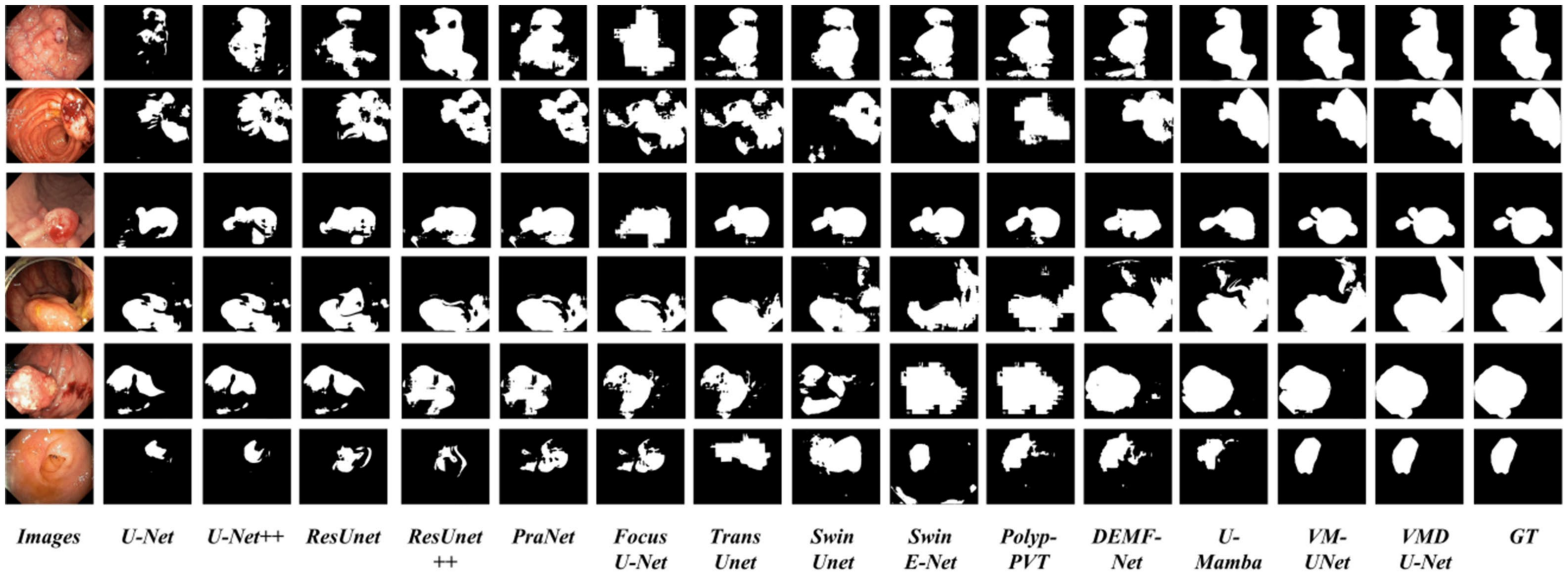
Partial visualization of segmentation results on the CVC-ClinicDB dataset.

In order to analyze VMDU-Net from a subjective perspective, this study presented segmentation results of various algorithms on the Kvasir-SEG and CVC-ClinicDB datasets in Figures 7, 8, respectively. These images not only reveal the performance differences among the algorithms in the polyp segmentation task but also highlight their respective strengths and weaknesses.

As shown in Figures 7, 8, convolutional neural network-based algorithms, such as U-Net and ResUnet, exhibit common issues of missed and false detections, triggering noticeable holes or artifacts in the segmentation results and overall poor performance. This not only affects the accuracy of the results but also diminishes the reliability of the models in practical applications. In contrast, Transformer-based algorithms and our Mamba algorithm demonstrate strong semantic consistency in segmentation tasks. These models can more effectively capture complex structures and details within images, significantly reduce both missed and false detections, and thus ensure the integrity and accuracy of the segmentation results. Notably, our proposed VMDU-Net yields segmentation results closest to the ground truth (GT), highlighting its superiority in handling complex images and extracting relevant features. Based on comparative analysis, this study has clearly observed the exceptional performance of VMDU-Net in polyp segmentation, and validated its potential and application value in medical image segmentation.

**Figure 9:**
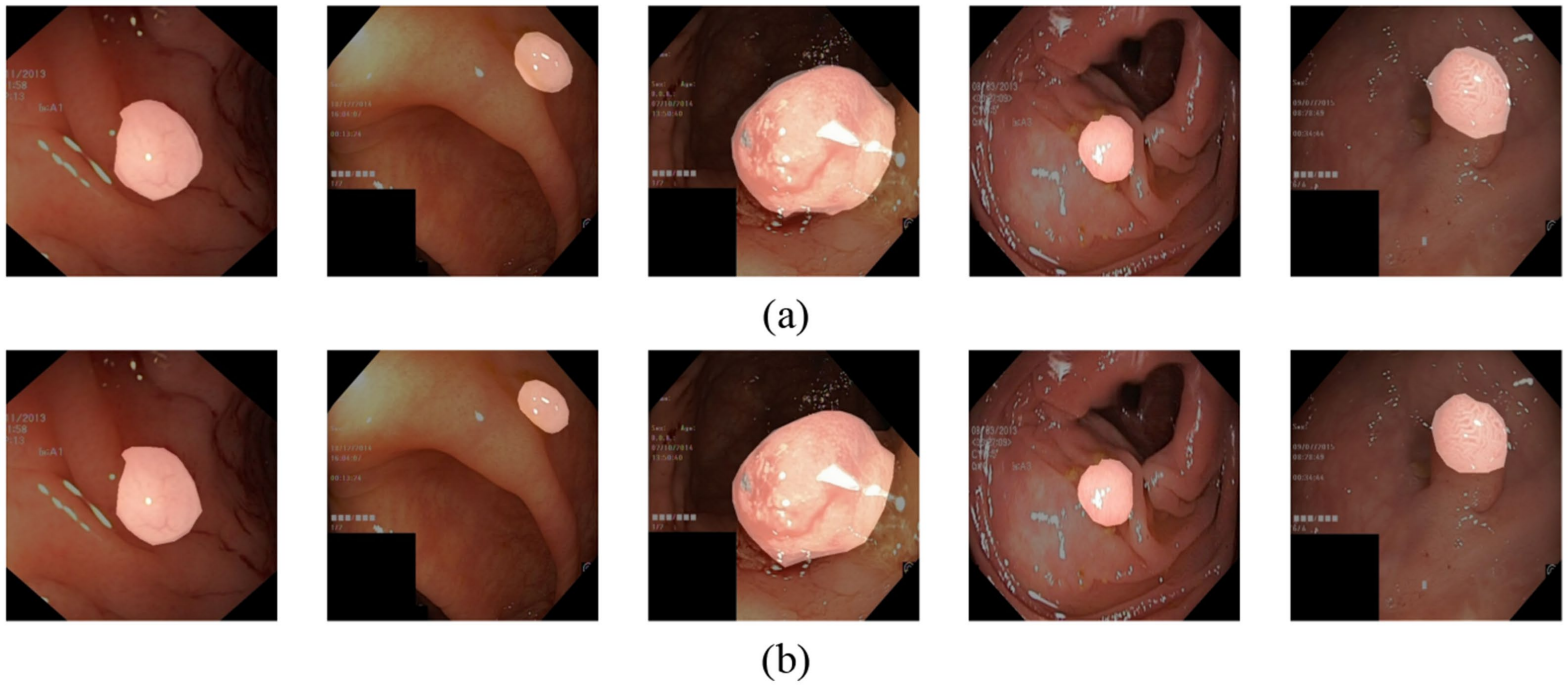
The prediction result of VMDU-Net. **(a)** Ground truth. **(b)** Prediction result of VMDU-Net.

In Figure 9, we fuse the segmentation predictions of VMDU-Net with the original images and their corresponding ground truth for visualization. The segmentation predictions of VMDU-Net closely match the polyp regions. Our proposed VMDU-Net generates results that align most closely with the GT, especially in fitting edge details, highlighting its advantages in handling polyp images and extracting relevant features.

## 5 Discussion

### 5.1 Finding

Through effective use of a dual-encoder architecture and a feature fusion mechanism, the proposed VMDU-Net network demonstrates outstanding performance in polyp segmentation tasks. In addition, VMDU-Net combines the Cross Shape Transformer (CST) with the Vision Mamba Encoder (VME) to capture long-range dependencies effectively and enhance the semantic understanding of the model. The innovative aspect of this design lies in CST’s utilization of a Cross Shape Self-Attention mechanism (CSA) for more efficient feature extraction, while VME focuses on capturing local features. The combination of these components enables the model to excel in handling complex medical images.

#### 5.1.1 Balancing long-range dependencies and local features

The dual-encoder design of VMDU-Net allows the model to rapidly capture long-range dependencies between pixels in the early stages of training. In the later stages, CST further explores deeper long-range dependencies. This process helps the model understand the global structure of images and maintain high-precision segmentation in local regions. The effectiveness of this structural design is reflected in the experimental results, where VMDU-Net outperforms existing mainstream segmentation algorithms across multiple public datasets.

#### 5.1.2 Effectiveness of feature fusion

The Mamba-Transformer-Merge (MTM) module plays a critical role in feature fusion. By effectively integrating features from CST and VME, the MTM module enhances the feature representation capabilities of the model and improves segmentation accuracy. As indicated by the experimental results, compared to traditional feature fusion methods, the MTM module better captures spatial location information in images, thereby enhancing segmentation precision and robustness.

#### 5.1.3 Comparison with existing methods

In comparison to other mainstream segmentation networks such as U-Net and ResUnet, VMDU-Net exhibits higher segmentation accuracy across multiple datasets. Notably, VMDU-Net demonstrates superior performance in handling complex polyp images, primarily due to its unique encoder design and feature fusion strategy. By incorporating Depthwise Separable Convolution as an auxiliary module during training, the model provides local bias information, which further improves convergence speed and segmentation effectiveness.

### 5.2 Limitations analysis

Although the promising results indicate that VMDU-Net performs well in polyp segmentation tasks, there are several limitations. First, while the datasets used (Kvasir-SEG, CVC-ClinicDB, EndoScene, CVC-ColonDB, and ETIS) are representative, they may vary in image quality, size, and annotation standards, potentially influencing the generalization ability of the model. Additionally, sample imbalance in certain datasets could hinder the model’s learning effectiveness for specific types of polyps.

Despite that VMDU-Net has achieved favorable performance, its complexity and computational demands may restrict its application in real clinical settings, particularly in real-time medical image analysis. Furthermore, model performance is highly dependent on hyperparameter selection, and optimal combinations may not always be identified. The findings of this study are primarily based on internal evaluations, but lack validation with real clinical data, limiting the practical applicability of the model.

Lastly, though all datasets have been annotated by experts, subjectivity among different annotators may lead to inconsistencies in labeling, further influencing the model training and evaluation. Therefore, future research should focus on issues such as dataset diversity, sample balance, model simplification, and external validation so as to enhance the clinical applicability of the model.

## 6 Conclusion

To conclude, this study presents a novel dual-encoder multi-scale feature fusion network, VMDU-Net, for the automated segmentation of colorectal polyps. By integrating the Vision Mamba component with the Cross-Shape Transformer, VMDU-Net effectively captures long-range dependencies and complex features associated with polyps. Experimental results indicate that our approach surpasses current state-of-the-art (SOTA) algorithms on both the Kvasir-SEG and CVC-ClinicDB datasets, attaining Dice coefficients of 0.934 and 0.951, respectively. The efficiency and accuracy of VMDU-Net in polyp segmentation significantly enhance the reliability of automated processes and provide strong technical support for the early detection and prevention of colorectal cancer. Furthermore, the Cross-Shape Transformer is specifically designed to utilize cross-shaped regions as tokens, effectively overcoming the limitations of conventional self-attention mechanisms in modeling long-range dependencies. Additionally, the Mamba-Transformer-Merge module contributes to improved segmentation accuracy by merging features from both encoders.

Future research can explore the application potential of VMDU-Net in other medical image segmentation tasks and continuously optimize the model architecture to address more complex clinical scenarios. Overall, VMDU-Net provides an innovative solution in the field of polyp segmentation and demonstrates significant clinical application value.

## Data Availability

The datasets presented in this study can be found in online repositories. The names of the repository/repositories and accession number(s) can be found at: https://datasets.simula.no/kvasir-seg/; https://polyp.grand-challenge.org/CVCClinicDB/; http://vi.cvc.uab.es/colon-qa/cvccolondb/; http://adas.cvc.uab.es/endoscene; https://paperswithcode.com/sota/medical-image-segmentation-on-etis.

## References

[ref1] Agarwal R. Ghosal P. Sadhu A. K. Murmu N. Nandi D. (2024). Multi-scale dual-channel feature embedding decoder for biomedical image segmentation. Comput. Methods Prog. Biomed. 257:108464. doi: 10.1016/j.cmpb.2024.108464, PMID: 39447437

[ref2] Alexey D. (2020). An image is worth 16x16 words: transformers for image recognition at scale. arXiv [Preprint]. *arXiv: 2010.11929*. doi: 10.48550/arXiv.2010.11929

[ref3] Banik D. Roy K. Bhattacharjee D. Nasipuri M. Krejcar O. (2020). Polyp-net: a multimodel fusion network for polyp segmentation. IEEE Trans. Instrum. Meas. 70, 1–12. doi: 10.1109/TIM.2020.3015607, PMID: 33776080

[ref4] Bernal J. Sánchez F. J. Fernández-Esparrach G. Gil D. Rodríguez C. Vilariño F. (2015). WM-DOVA maps for accurate polyp highlighting in colonoscopy: validation vs. saliency maps from physicians. Comput. Med. Imaging Graph. 43, 99–111. doi: 10.1016/j.compmedimag.2015.02.007, PMID: 25863519

[ref5] Cao H. Wang Y. Chen J. Jiang D. Zhang X. Tian Q.. (2022) Swin-unet: Unet-like pure transformer for medical image segmentation. In European conference on computer vision, pp. 205–218: Springer

[ref6] Cao X. Yu H. Yan K. Cui R. Guo J. Li X.. (2024). DEMF-net: a dual encoder multi-scale feature fusion network for polyp segmentation. Biomed. Signal Process. Control 96:106487. doi: 10.1016/j.bspc.2024.106487

[ref7] Chen J. Lu Y. Yu Q. Luo X. Adeli E. Wang Y.. (2021). Transunet: transformers make strong encoders for medical image segmentation. arXiv [Preprint]. *arXiv:2102.04306*. doi: 10.1016/j.media.2024.103280

[ref8] Diakogiannis F. I. Waldner F. Caccetta P. Wu C. (2020). ResUNet-a: A deep learning framework for semantic segmentation of remotely sensed data. ISPRS J. Photogramm. Remote Sens. 162, 94–114. doi: 10.1016/j.isprsjprs.2020.01.013

[ref9] Dong B. Wang W. Fan D.-P. Li J. Fu H. Shao L. (2021). Polyp-pvt: polyp segmentation with pyramid vision transformers. arXiv [Preprint]. *arXiv:2108.06932*. doi: 10.26599/AIR.2023.9150015

[ref10] Duc N. T. Oanh N. T. Thuy N. T. Triet T. M. Dinh V. S. (2022). Colonformer: an efficient transformer based method for colon polyp segmentation. IEEE Access 10, 80575–80586. doi: 10.1109/ACCESS.2022.3195241

[ref11] Fan D.-P. Ji G.-P. Zhou T. Chen G. Fu H. Shen J.. (2020) Pranet: parallel reverse attention network for polyp segmentation. In International conference on medical image computing and computer-assisted intervention, pp. 263–273: Springer

[ref12] Fan C. Yu H. Wang L. Huang Y. Wang L. Jia X. (2024). SliceMamba for medical image segmentation. arXiv [E-prints]. *arXiv:2407.08481*. doi: 10.1109/JBHI.2025.3564381

[ref13] Ghosal P. Roy A. Agarwal R. Purkayastha K. Sharma A. L. Kumar A. (2024). Compound attention embedded dual channel encoder-decoder for ms lesion segmentation from brain MRI. Multimed. Tools Appl. 177, 1–33. doi: 10.1007/s11042-024-20416-3

[ref14] Gu A. Dao T. (2023). Mamba: linear-time sequence modeling with selective state spaces. arXiv [Preprint]. *arXiv:2312.00752*.

[ref15] Guo X. Lin X. Yang X. Yu L. Cheng K.-T. Yan Z. (2024). UCTNet: uncertainty-guided CNN-transformer hybrid networks for medical image segmentation. Pattern Recogn. 152:110491. doi: 10.1016/j.patcog.2024.110491

[ref16] Guo X. Yang C. Liu Y. Yuan Y. (2020). Learn to threshold: Thresholdnet with confidence-guided manifold mixup for polyp segmentation. IEEE Trans. Med. Imaging 40, 1134–1146. doi: 10.1109/TMI.2020.3046843, PMID: 33360986

[ref17] Gupta M. Mishra A. (2024). A systematic review of deep learning based image segmentation to detect polyp. Artif. Intell. Rev. 57:7. doi: 10.1007/s10462-023-10621-1

[ref18] Haggar F. A. Boushey R. P. (2009). Colorectal cancer epidemiology: incidence, mortality, survival, and risk factors. Clin. Colon Rectal Surg. 22, 191–197. doi: 10.1055/s-0029-1242458, PMID: 21037809 PMC2796096

[ref19] Isensee F. Petersen J. Klein A. Zimmerer D. Jaeger P. F. Kohl S.. (2018) Nnu-net: self-adapting framework for u-net-based medical image segmentation. *arXiv* [Preprint]. *arXiv:1809.10486*. doi: 10.1038/s41592-020-01008-z

[ref20] Jha D. Smedsrud P. H. Riegler M. A. Halvorsen P. De Lange T. Johansen D.. (2019) Kvasir-seg: A segmented polyp dataset. In International conference on multimedia modeling, pp. 451–462: Springer International Publishing Cham

[ref21] Jha D. Smedsrud P. H. Riegler M. A. Johansen D. De Lange T. Halvorsen P.. (2019) Resunet++: an advanced architecture for medical image segmentation. In 2019 IEEE international symposium on multimedia (ISM), (pp. 225–2255): IEEE

[ref22] Jha D. Tomar N. K. Sharma V. Bagci U. (2024) TransNetR: transformer-based residual network for polyp segmentation with multi-center out-of-distribution testing. In Medical imaging with deep learning, pp. 1372–1384: PMLR

[ref23] Jia X. Xing X. Yuan Y. Xing L. Meng M. Q.-H. (2019). Wireless capsule endoscopy: a new tool for cancer screening in the colon with deep-learning-based polyp recognition. Proc. IEEE 108, 178–197.

[ref24] Kim T. Lee H. Kim D. (2021) Uacanet: uncertainty augmented context attention for polyp segmentation. In Proceedings of the 29th ACM international conference on multimedia, pp. 2167–2175

[ref25] Li Y. Yao T. Pan Y. Mei T. (2022). Contextual transformer networks for visual recognition. IEEE Trans. Pattern Anal. Mach. Intell. 45, 1489–1500. doi: 10.1109/TPAMI.2022.3164083 35363608

[ref26] Lin A. Chen B. Xu J. Zhang Z. Lu G. Zhang D. (2022). Ds-transunet: dual swin transformer u-net for medical image segmentation. IEEE Trans. Instrum. Meas. 71, 1–15.

[ref27] Liu Z. Lin Y. Cao Y. Hu H. Wei Y. Zhang Z.. (2021). Swin transformer: hierarchical vision transformer using shifted windows. In Proceedings of the IEEE/CVF international conference on computer vision, pp. 10012–10022

[ref28] Liu J. Yang H. Zhou H.-Y. Xi Y. Yu L. Li C.. (2024) Swin-umamba: mamba-based unet with imagenet-based pretraining. In International conference on medical image computing and computer-assisted intervention, pp. 615–625: Springer Nature Switzerland Cham

[ref29] Ma J. Li F. Wang B. (2024). U-mamba: enhancing long-range dependency for biomedical image segmentation. arXiv [Preprint]. *arXiv:2401.04722*

[ref30] Narayanan M. (2023). SENetV2: aggregated dense layer for channelwise and global representations. arXiv [Preprint]. *arXiv:2311.10807*.

[ref31] Oktay O. Schlemper J. Folgoc L. L. Lee M. Heinrich M. Misawa K.. (2018). Attention u-net: learning where to look for the pancreas. arXiv [Preprint]. *arXiv:1804.03999*

[ref32] Park K.-B. Lee J. Y. (2022). SwinE-net: hybrid deep learning approach to novel polyp segmentation using convolutional neural network and Swin transformer. J. Comput. Design Eng. 9, 616–632. doi: 10.1093/jcde/qwac018

[ref33] Ratheesh A. Soman P. Nair M. R. Devika R. Aneesh R. (2016) Advanced algorithm for polyp detection using depth segmentation in colon endoscopy. In 2016 international conference on communication systems and networks (ComNet), pp. 179–183: IEEE

[ref34] Ronneberger O. Fischer P. Brox T. (2015) U-net: convolutional networks for biomedical image segmentation. In Medical image computing and computer-assisted intervention–MICCAI 2015: 18th international conference, Munich, Germany, October 5–9, 2015, proceedings, part III 18, pp. 234–241: Springer International Publishing

[ref35] Ruan J. Li J. Xiang S. (2024). Vm-unet: Vision mamba unet for medical image segmentation. arXiv [Preprint]. *arXiv:2402.02491*

[ref36] Sanderson E. Matuszewski B. J. (2022) FCN-transformer feature fusion for polyp segmentation. In Annual conference on medical image understanding and analysis, pp. 892–907: Springer

[ref37] Sasmal P. Bhuyan M. K. Dutta S. Iwahori Y. (2022). An unsupervised approach of colonic polyp segmentation using adaptive Markov random fields. Pattern Recogn. Lett. 154, 7–15. doi: 10.1016/j.patrec.2021.12.014

[ref38] Silva J. Histace A. Romain O. Dray X. Granado B. (2014). Toward embedded detection of polyps in wce images for early diagnosis of colorectal cancer. Int. J. Comput. Assist. Radiol. Surg. 9, 283–293. doi: 10.1007/s11548-013-0926-3 24037504

[ref39] Sun X. Zhang P. Wang D. Cao Y. Liu B. (2019) Colorectal polyp segmentation by U-net with dilation convolution. In 2019 18th IEEE international conference on machine learning and applications (ICMLA), pp. 851–858: IEEE

[ref40] Tajbakhsh N. Gurudu S. R. Liang J. (2015). Automated polyp detection in colonoscopy videos using shape and context information. IEEE Trans. Med. Imaging 35, 630–644. doi: 10.1109/TMI.2015.2487997, PMID: 26462083

[ref41] Tang H. Huang G. Cheng L. Yuan X. Tao Q. Chen X.. (2024). RM-UNet: UNet-like mamba with rotational SSM module for medical image segmentation. SIViP 18, 8427–8443. doi: 10.1007/s11760-024-03484-8

[ref42] Tomar N. K. Shergill A. Rieders B. Bagci U. Jha D. (2022). TransResU-net: transformer based ResU-net for real-time colonoscopy polyp segmentation. arXiv [Preprint]. *arXiv:2206.08985*. 10.1109/EMBC40787.2023.10340572 38083589

[ref43] Vaswani A. (2017). Attention is all you need. Adv. Neural Inf. Proces. Syst. 14.

[ref44] Vázquez D. Bernal J. Sánchez F. J. Fernández-Esparrach G. López A. M. Romero A.. (2017). A benchmark for endoluminal scene segmentation of colonoscopy images. J. Healthcare Eng. 2017, 1–9. doi: 10.1155/2017/4037190, PMID: 29065595 PMC5549472

[ref45] Waleffe R. Byeon W. Riach D. Norick B. Korthikanti V. Dao T.. (2024) An empirical study of mamba-based language models. arXiv [Preprint]. *arXiv:2406.07887*

[ref46] Wang H. Cao P. Wang J. Zaiane O. R. (2022) Uctransnet: rethinking the skip connections in u-net from a channel-wise perspective with transformer. In Proceedings of the AAAI conference on artificial intelligence, Vol. 36, pp. 2441–2449

[ref47] Wang H. Xie S. Lin L. Iwamoto Y. Han X.-H. Chen Y.-W.. (2022) Mixed transformer u-net for medical image segmentation. In ICASSP 2022–2022 IEEE international conference on acoustics, speech and signal processing (ICASSP), pp. 2390–2394): IEEE

[ref48] Wu H. Zhao Z. Wang Z. (2023). META-Unet: multi-scale efficient transformer attention Unet for fast and high-accuracy polyp segmentation. IEEE Trans. Autom. Sci. Eng. 53.

[ref49] Xiao B. Hu J. Li W. Pun C.-M. Bi X. (2024). CTNet: contrastive transformer network for polyp segmentation. IEEE Trans. Cybern. 50. 10.1109/TCYB.2024.3368154 38470573

[ref50] Xie Y. Zhang J. Shen C. Xia Y. (2021) Cotr: efficiently bridging cnn and transformer for 3d medical image segmentation. In Medical image computing and computer assisted intervention–MICCAI 2021: 24th international conference, Strasbourg, France, September 27–October 1, 2021, Proceedings, Part III 24, pp. 171–180: Springer

[ref51] Xing Z. Ye T. Yang Y. Liu G. Zhu L. (2024) Segmamba: long-range sequential modeling mamba for 3d medical image segmentation. In International conference on medical image computing and computer-assisted intervention, pp. 578–588: Springer Nature Switzerland Cham

[ref52] Yeung M. Sala E. Schönlieb C.-B. Rundo L. (2021). Focus U-net: a novel dual attention-gated CNN for polyp segmentation during colonoscopy. Comput. Biol. Med. 137:104815. doi: 10.1016/j.compbiomed.2021.104815, PMID: 34507156 PMC8505797

[ref53] Zhang M. Chen Z. Ge Y. Tao X. (2024). HMT-UNet: a hybird mamba-transformer vision UNet for medical image segmentation. arXiv [Preprint]. *arXiv:2408.11289*

[ref54] Zhang R. Li G. Li Z. Cui S. Qian D. Yu Y. (2020) Adaptive context selection for polyp segmentation. In Medical image computing and computer assisted intervention–MICCAI 2020: 23rd international conference, Lima, Peru, October 4–8, 2020, Proceedings, Part VI 23, pp. 253–262: Springer

[ref55] Zhang Y. Liu H. Hu Q. (2021). Transfuse: fusing transformers and cnns for medical image segmentation. In Medical image computing and computer assisted intervention–MICCAI 2021: 24th international conference, Strasbourg, France, September 27–October 1, 2021, proceedings, Part I 24, pp. 14–24: Springer

[ref56] Zhou Z. Rahman Siddiquee M. M. Tajbakhsh N. Liang J. (2018) Unet++: a nested u-net architecture for medical image segmentation. In Deep learning in medical image analysis and multimodal learning for clinical decision support: 4th international workshop, DLMIA 2018, and 8th international workshop, ML-CDS 2018, held in conjunction with MICCAI 2018, Granada, Spain, September 20, 2018, proceedings 4, pp. 3–11: Springer International Publishing 10.1007/978-3-030-00889-5_1 PMC7329239 32613207

[ref57] Zhu L. Liao B. Zhang Q. Wang X. Liu W. Wang X. (2024) Vision mamba: efficient visual representation learning with bidirectional state space model. arXiv [Preprint]. *arXiv:2401.09417*.

[ref58] Zhu X. Wang W. Zhang C. Wang H. (2025). Polyp-mamba: a hybrid multi-frequency perception gated selection network for polyp segmentation. Inf. Fusion 115:102759. doi: 10.1016/j.inffus.2024.102759

